# Disease Gene Characterization through Large-Scale Co-Expression Analysis

**DOI:** 10.1371/journal.pone.0008491

**Published:** 2009-12-31

**Authors:** Allen Day, Jun Dong, Vincent A. Funari, Bret Harry, Samuel P. Strom, Dan H. Cohn, Stanley F. Nelson

**Affiliations:** 1 Department of Human Genetics, Molecular Biology Institute, David Geffen School of Medicine, University of California Los Angeles, Los Angeles, California, United States of America; 2 Cedars-Sinai Medical Center, Medical Genetics Institute, Los Angeles, California, United States of America; 3 Department of Pediatrics, David Geffen School of Medicine, University of California Los Angeles, Los Angeles, California, United States of America; 4 Department of Psychiatry, David Geffen School of Medicine, University of California Los Angeles, Los Angeles, California, United States of America; Baylor College of Medicine, United States of America

## Abstract

**Background:**

In the post genome era, a major goal of biology is the identification of specific roles for individual genes. We report a new genomic tool for gene characterization, the UCLA Gene Expression Tool (UGET).

**Results:**

Celsius, the largest co-normalized microarray dataset of Affymetrix based gene expression, was used to calculate the correlation between all possible gene pairs on all platforms, and generate stored indexes in a web searchable format. The size of Celsius makes UGET a powerful gene characterization tool. Using a small seed list of known cartilage-selective genes, UGET extended the list of known genes by identifying 32 new highly cartilage-selective genes. Of these, 7 of 10 tested were validated by qPCR including the novel cartilage-specific genes *SDK2* and *FLJ41170*. In addition, we retrospectively tested UGET and other gene expression based prioritization tools to identify disease-causing genes within known linkage intervals. We first demonstrated this utility with UGET using genetically heterogeneous disorders such as Joubert syndrome, microcephaly, neuropsychiatric disorders and type 2 limb girdle muscular dystrophy (LGMD2) and then compared UGET to other gene expression based prioritization programs which use small but discrete and well annotated datasets. Finally, we observed a significantly higher gene correlation shared between genes in disease networks associated with similar complex or Mendelian disorders.

**Discussion:**

UGET is an invaluable resource for a geneticist that permits the rapid inclusion of expression criteria from one to hundreds of genes in genomic intervals linked to disease. By using thousands of arrays UGET annotates and prioritizes genes better than other tools especially with rare tissue disorders or complex multi-tissue biological processes. This information can be critical in prioritization of candidate genes for sequence analysis.

## Introduction

The completion of the human genome, elucidation of most protein coding genes, and development of new tools for the assessment of genomic variation and regulation, have greatly facilitated our ability to identify specific genes and gene variants involved in diverse human traits. As information accumulates, there is substantial promise that advances in biological understanding will come through integrative approaches that combine genomic data acquired from many sources [1], [2], [3], [4]. One of the largest sources of information is derived from genome-wide gene expression data made possible through academic and commercial efforts [5], [6].

Integrating whole genome linkage data with whole genome expression data may help annotate and prioritize genes for mutation analysis in disease linkage or genome wide association study intervals [7], [8]. Unlike information based approaches for prioritizing genes in intervals such as peer reviewed literature and Gene Ontology (e.g. Prospector [9], GeneWanderer [10], SUSPECTS [11], PosMed [12], GeneSniffer [13], etc.), using gene expression data may rank all the genes in an interval even those with little characterization (e.g. novel genes). Current tools (GeneDistiller [14] and Endeavor [15], ToppGene Suite [16]), which incorporate whole gene expression data all use the well annotated but limited discrete datasets like the well known Gene Expression Atlas dataset from Novartis which includes a genome wide expression survey of 40 normal tissues [17]. Although the utility of gene-gene co-expression patterns to aggregate genes of similar function has been demonstrated [18], [19], [20], [21], [22], [23], individual microarray studies frequently suffer from the lack of statistical power because of the relatively small numbers of samples observed relative to the number of genes measured [24] or the inability and great expense needed to capture all possible biological states in one experiment. Thus, efforts at establishing larger aggregates of data for meta-analysis have been pursued. For instance, a large collection of cancer related microarray studies has been organized at Oncomine Research [25], which has led to novel gene discoveries not robustly identified in the individual datasets [26], [27]. Additionally, Genesapiens has pooled the largest group of consistently well-annotated diverse microarray data in the public domain. It contains both normal and disease tissue datasets and can be queried for gene expression or gene-gene co-expression networks that can be presented in graphical format. Though the effort to annotate 17,330 human genes across 9,783 different samples collected from the public domain is powerful, it remains an incomplete dataset with many tissues, developmental states and genes not present in the database. Other large gene expression databases like Genelogic's gene expression database may be more robust but are unavailable to the public [28].

In general, the common wisdom within the genomics community is that annotation information is key to the reuse of these data [29], [30], [31]. While experimental annotation provides additional power in data analysis, we believe that queries using only small focused annotated datasets may be limiting. Therefore, in exchange for complete absence of metadata we have created a robust gene-gene co-expression network which we demonstrate can outperform more focused, but smaller scale, experiments in many cases. We constructed this network using filtered and co-normalized data from Celsius, the largest data warehouse of Affymetrix microarray data [32]. Celsius has accumulated more than 150,000 co-normalized microarrays across the various Affymetrix array designs, so is among the most powerful resources for expression analysis. As an example of the scale, as of August 2008 more than 12,000 U133_2.0 arrays were available, which is approximately four times larger than Genesapiens.

Here we describe the creation of a generic, web accessible tool that we call UGET (UCLA Gene Expression Tool; http://genome.ucla.edu/projects/UGET) that taps directly into the vast amount of data within Celsius. In brief, we measure the correlation of all genes with all genes within a given array platform and provide a rapid search tool to retrieve signals of interest. Three general examples of the use of this tool are presented. First, UGET is used to identify a series of genes with cartilage-selective expression, which are excellent candidates for skeletal dysplasia mutation bearing genes extending previous work [33]. This same approach can be implemented to identify gene lists relevant to other disease/traits/tissue functions. Second, we apply the large-scale human gene-gene correlation analysis to a retrospective analysis of prioritization of individual genes for mutation analysis within various linkage regions in five different disease models: muscular dystrophy, microcephaly, Joubert Syndrome, skeletal dysplasia, and neuropsychiatric disorders. We compare these results to results obtained by other tools which also use whole genome gene expression to prioritize genes. While we demonstrate the successful use of the tool for humans, we note that the tool includes data for 14 species on 41 different array platforms.

## Results

Our aim was to create a tool that permits scientists to explore the data available within Celsius and demonstrate the utility of these data in human disease gene identification as a general proxy of the information within the dataset. To do this we created data matrices of gene-gene correlations and demonstrate two methods to simply mine the matrix of correlation coefficients. For these demonstrations we use the Affymetrix HG-U133_Plus_2 array design. We use in these analyses probeset 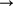 gene symbol mappings available from NetAffx [34] and probeset 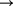 genome alignments available from the UCSC Genome Browser [35] and exclude all other information about the microarray experiments that were performed. In the prediction of gene function we utilized human-reviewed Gene Ontology (GO) Biological Process (BP) codes, as available from Bioconductor [36], [37]. In all cases, metadata about the biological samples, sample treatments, and other conditions of the original experiments were omitted from our analyses in order to demonstrate the power of the approach in the absence of annotation data.

**Figure 1:**
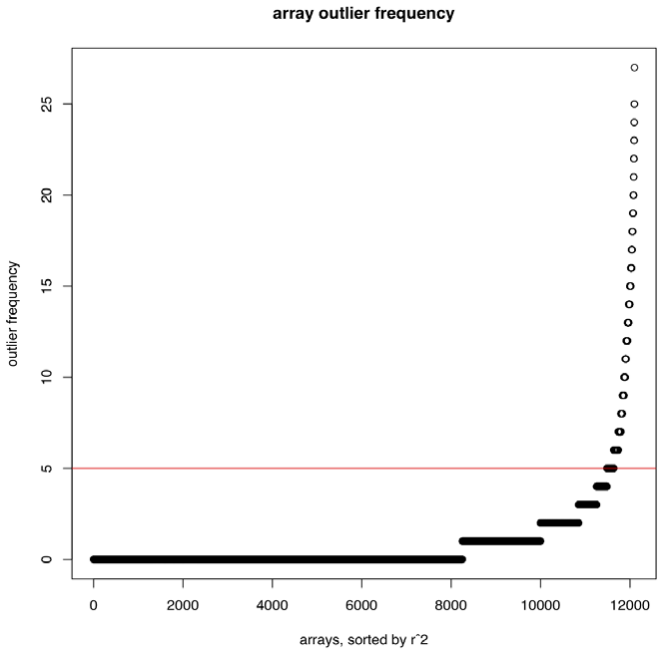
Regressions of control probesets reveal aberrant arrays. Multiple regressions were performed for all 62 HG-U133_Plus_2 control probesets. Arrays (x-axis) are plotted versus the fraction of observations with regression residual 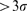 (y-axis). A dashed horizontal line indicates a cutoff above which arrays are omitted from analysis.

The first step of the array processing demands some level of removal of poorly performing arrays, as systematic high and low signals that are highly correlated would dominate the results. As an example of this filtering process, from 12,826 arrays available within Celsius and performed on the HG-U133_Plus_2 arrays 711 arrays were removed as having signal that was 3 standard deviations below the mean of the whole group and 15 were removed for having signal 3 standard deviations above the mean of the whole group. In addition, 464 arrays were removed as the correlations between 62 probesets, which are intended to be measuring the same doped-in transcript, had high variability (Figure 1). Thus, 11,636 arrays remained for analyses, which were processed for probeset intensities using RMA [38] as implemented within Celsius as previously described [32]. From these 11,636 arrays we calculated the Pearson correlation coefficient for every pair of 54,675 probesets yielding a 54,675×54,675 correlation matrix, denoted 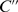. These data are stored online and are accessible in bulk (http://genome.ucla.edu/u/projects/UGET/matrices) or are searchable through a web application called UGET. Each array platform was processed in a similar fashion. The July 2008 freeze is used for all of the reported analyses here.

### Functional Assignment of Genes

**Figure 2:**
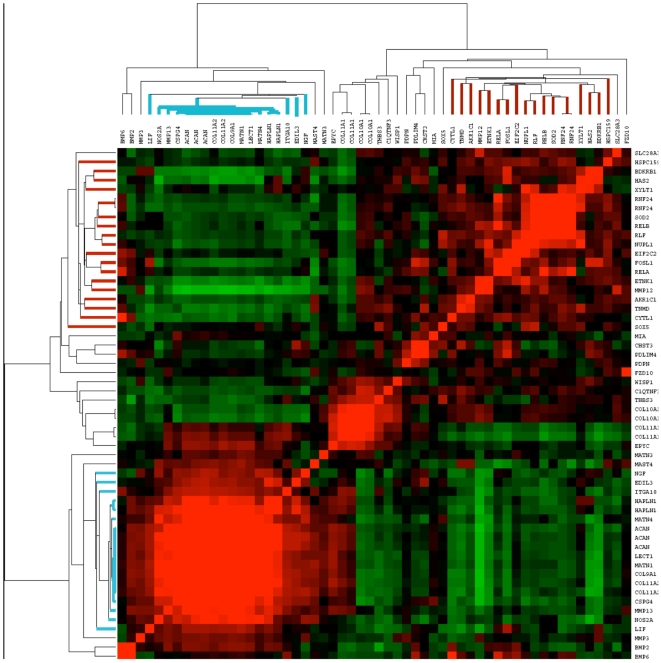
Analysis of cartilage-selective probesets previously identified on U133A platform using two-way clustering of gene-gene correlation data. Gene-gene correlations were identified for all cartilage-selective probesets previously identified on the U133A platform [6]. Dendrograms from two-way clustering of the median-centered correlation data suggest three distinct expression patterns. The strongest node (blue) was selected as a cartilage profile for further expansion by seeding the UGET.

We first attempted to determine if the agglomerated data and gene-gene correlation matrix could be applied to a well studied and useful effort to identify genes with cartilage selective expression, and identify novel genes not obvious in more directed prior experimentation. Funari and colleagues compared gene expression in developing cartilage with a set of non-cartilage tissues to identify genes with a pattern of high expression in cartilage and little or no expression in non-cartilage tissues [33]. These data have already been used for successful candidate gene identification for skeletal dysplasias and facilitated identification of genes causing two skeletal dysplasia phenotypes [7], [8]. To illustrate the use of UGET in this way, we attempted to expand the gene list of a subset of cartilage-selective genes [23], [33]. The original list of 161 genes was identified on three platforms and was enriched for genes that when mutated lead to skeletal anomalies. A subset of these genes (52 genes; 58 probesets) identified on the U133A platform was selected to assess the utility and validity of UGET. Gene-gene correlation data from the 58 probesets were median centered in column and row to easily visualize differences, then subjected to 2-way hierarchical clustering (Figure 2). Within the 11,636 arrays, genes from the original cartilage-selective list clustered into three distinct groups, suggesting three different patterns of gene expression [33]. Interestingly, one cluster of genes (highlighted in blue in Figure 2) contains most of the well known cartilage-specific proteins and mutations in 14/18 genes in this cluster can cause skeletal anomalies in humans or mice. Of the 4/18 genes (*LIF*, *CSPG4*, *EDIL3*, and *MATN4*) not associated with skeletal anomalies, two have not been characterized in mouse models. They also contain the strongest correlations and therefore most homogenous expression pattern. Therefore, genes with the highest correlation at the center of this node were then used as a primary seed to search for additional genes with the same expression pattern. This first step in the assembly of the gene expression patterns with Celsius is to identify the probesets with the highest correlations of this seed list of known, related genes (referred to as a *profile*). Next, the mean correlation coefficient to the profile (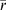) was calculated for each gene across the genome that was present on the array. Finally, we rank-ordered the set of all probesets and the set of the initially selected probesets by 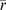.

Using the default setting, the one hundred genes with the highest correlation to the seed profile were returned (Table S1). 44 of these genes (56 probesets) were identified in a previous supervised analysis in Funari, 2007 et. al., which used well-annotated arrays. These genes are indicated by the column “CV and fold change” in the Table S1. These results suggest that even without metadata, the UGET analysis identifies a comparable gene list. In fact, some of the differences among the lists may be attributed to the stringent filtering in the supervised analysis, the emphasis of the statistical approaches, the use of different platforms, and the limited number of tissues and arrays surveyed in the classical preliminary study. To illustrate this, when our list was compared to the supervised 2-class SAM analysis performed on the similar U133 2.0 platform, 76 probesets (59 genes) were identified in the top 200 ranked probesets. Assuredly, there was a dramatic enrichment of probes for genes that have already been associated with skeletal defects in mouse or man using the UGET provided hyperlinks for the associated genes in Online Mendelian Inheritance in Man (OMIM) and Mouse Genome Database (MGD) databases [39], [40]. Although only 44/100 probesets were associated with gene targeted mouse phenotypes, 23 of these were associated with genes that appear to be involved in normal fetal cartilage developmental in mice. Additionally, 10 more probesets represented genes associated with skeletal defects but have not yet been identified as associated with fetal cartilage development (e.g. Bone, Adult Cartilage, other Skeletal phenotypes). Most of the probesets (54) could not be associated with a single mouse model, illustrating at least in part how relatively uncharacterized the cartilage genome is. It is likely that many of these and other skeletal probesets will later be associated with genes with critical roles in cartilage development in man and/or mice. An example of this is the lack of an obvious cartilage development phenotype in TRPV4 targeted mice, however gain of function mutations in TRPV4 can cause *Brachyolmia in humans* [7]. Additionally, mutations in COL9A2 cause epiphyseal dysplasia [41], yet there is not yet a targeted mouse model for this gene. Interestingly, the gene-gene correlations identified 32 genes not previously defined as cartilage-selective [33] (gene rows are highlighted in Table S1). Some of these newly identified genes are well characterized as cartilage-selective and associated with one of a variety of forms of skeletal dysplasia (e.g., TRPV4, COL2A1, COMP, and COL9A3). In fact, although TRPV4 was not identified as cartilage-selective in the Funari et al. 2007 effort because of its low level of expression in cartilage, Rock and colleagues have recently characterized TRPV4 mutations as causative of brachyolmia [7]. To further validate these genes as cartilage-selective, qPCR was used to survey a subset for expression level in normal fetal cartilage and seven non-cartilage tissues (Table 1). Seven genes equally distributed in the ranked list (ca. every 1*0* genes) for which little functional annotation existed were used for validation. Top ranked and cartilage-selective ACAN was used as a positive control to illustrate a known cartilage-selective expression pattern while no-template reactions served to assess specificity. In brief, three of the seven genes appear to be relatively cartilage-selective, containing minimal if any expression in other tissues. Gross abnormal skeletal structure, decreased body length and growth retardation were reported in FZD9 targeted mouse (Fzd9^tm1Lex^) [40], while no functional characterization of SDK2 and FLJ41170 has yet been performed. Importantly, all of the genes demonstrated a cartilage-selective expression pattern, expressing on average 6.4 times more of the transcript than any non-cartilage tissue with little if any expression in non-cartilage tissues (∼Ct = 33). The strength of this tool is realized in this example. Unlike most of the well-characterized extracellular matrix proteins (e.g. ACAN) with high expression in cartilage (Ct = 24), this group of validated genes represented low expression in cartilage (ca. Ct = 29). The sensitivity of this analysis underscores the power of using a large array dataset for gene-gene correlation measures. In effect, the scale of the data reduces the otherwise substantial requirements for a minimum fold change filter often used to circumvent false positive detection when using small numbers of samples.

**Table 1:** Summary of qRT-PCR amplification of cartilage-selective genes in fetal cartilage and seven non-cartilage tissues.

		Ct			
Rank	Gene	Cart	NCart	Neg	Fold
4	*ACAN*	24	34	6	1024
23	*FZD9*	29		6	Unique
32	*SDK2*	31		7	Unique
47	*SLC39A14*	25	28		8
64	*PDE10A*	31	33	3	4
67	*SUSD5*	29	32	2	8
83	*TWSG1*	28	31		8
93	*FLJ41170*	31	33	4	4

Representative genes are listed in rank order of similarity to the human cartilage selective genes used in the seeding profile. Cart: mean Ct value for cartilage samples. NCart: Mean Ct value for the 7 non-cartilage samples. Neg: Number of non-cartilage tissues in which amplification was not detected at 35 cycles. Where no amplification was observed the maximum Ct value (i.e. 35) was used for calculations. Fold difference (Fold) is calculated from the difference in cartilage and non-cartilage Ct values.

Finally, mouse often provides a good model for many diseases and the findings from searching the human gene-gene correlations should be supported in the available mouse data. The mouse orthologues of the 14 human genes used as a profile of human cartilage were also used as a profile for mouse cartilage. Approximately, 50 percent of the human cartilage-selective genes (44 genes [54 probes]) were also identified in mouse as cartilage-selective. Within this group, twenty-two genes were identified which cause skeletal defects in mice or humans, which is a high enrichment of skeletal dysplasia genes. In aggregate, these results demonstrate the power of the unannotated expression data to identify genes of similar expression pattern and role in a disease process, which were validated in silico and in vitro.

### Disease Gene Identification

Typically, a genomic region is initially linked to Mendelian disease through the observation of regional haplotypes shared in excess within affected pedigrees, delineating a single genomic interval to be examined for causative mutations [42], [43]. These linkage regions are commonly up to 6–15 megabases (Mb) in size, and thus typically contain on the order of 100–300 genes, many of which lack any meaningful characterization. This complicates the disease gene discovery processes, and can stall progress significantly.

Often there are one to several different genes (outside a linked interval of interest) that, when mutated, lead to an identical or highly similar phenotype. For instance, Joubert Syndrome (JBST) is known to map to at least 7 different loci and Limb Girdle Muscular Dystrophy Type 2 (LGMD2) maps to at least 11 loci, and both will likely map to more. As additional disease genes for a given condition are identified, it is commonly observed that all the identified genes play critical roles in a shared biological process (BP), and when any one of the components of this process is disrupted it leads to the dysfunctional phenotype. Given that these genes are involved in the same BP, it is reasonable to assume that they will be co-expressed in similar tissues/cell lines or in response to similar exogenous treatments of cells that have been performed and assessed on genome-wide microarrays deposited in Celsius. Thus, we anticipate that there would be a net-positive correlation of expression levels among the diverse set of disease causing genes at different locations in the genome which all cause a similar disease when mutated. To demonstrate this we retrospectively assess known linkage intervals by rank ordering all genes over the linkage interval by their correlation coefficient with known disease causing genes (or a set of genes known to participate in the critical BP).

Our method was to assemble a list of genes 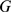 known to be associated with the disease or critical biological process. Each gene identifier 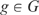 was mapped to the corresponding list of probesets on the HG-U133_Plus_2 array design. The list is denoted 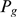, and we denote this mapping function as 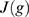. For each probeset 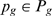, the genomic position was retrieved using the UCSC Genome Browser [35]. We then retrieved a list of probesets 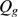, which aligned to the candidate interval 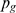, and we denote this mapping function as 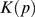. Next, for each probeset 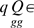 we calculated the mean correlation coefficient 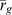 to 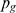 using 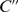, and we denote this as *L(q,C″)*. Finally, we rank order each gene within the interval by the maximum correlation coefficient of probes mapping back to gene symbol (max 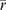).

**Figure 3:**
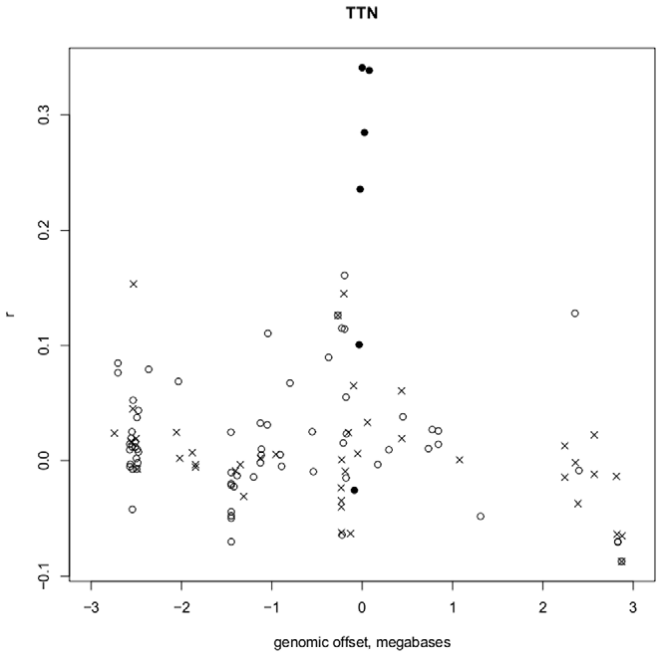
Gene correlations to a list of LMGD2-associated genes within a 6 megabase region surrounding the location of a known associated gene. The genomic position (x-axis) of probesets within a 6 megabase region centered at the location of *TTN*, a gene known to be associated with LMGD2, is plotted versus the Pearson correlation coefficient 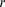 (y-axis) to a list of probesets targeting other genes known to be associated with LGMD2 (excluding *TTN*) across 11636 HG-U133_Plus_2 microarrays. Solid circles: probesets targeting *TTN*, 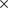: probesets that are for genes of unknown function and, open circles: probesets for known genes in interval.

We first applied our method to LGMD2, which is genetically heterogeneous. There are 11 genes known to be mutated that lead to LGMD2: *CAPN3, TCAP, TTN, SGCA, SGCB, SCGD, SGCG, POMT1, FKRP, TRIM32* and *DYSF* [44]. In order to determine if we could highlight the known gene of interest within the approximate linkage interval by using a profile created from the other known LGMD2 genes, we considered a 6 Mb interval centered at each of the 11 named genes above. All probesets designed to measure gene expression from the U133_2.0_Plus arrays within the 6 Mb genomic region were included for analysis. We calculated the mean correlation coefficient 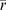 to the 10-gene LGMD2 profile for each probeset within each of the 11 regions, as we excluded any probesets targeting the gene that maps within the selected linkage region. For instance, in the titin (*TTN*) interval, all genes except *TTN* were used for the gene correlation calculation. In 55% (6/11) of the cases max 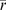 corresponded to the causal gene for LGMD2. In 2 of the 11 cases the causative gene was the second most highly correlated with the LGMD2 gene set, for three genes (*FKRP, POMT1*, and *DYSF*) there was poor correlation with the LGMD2 gene set. The high frequency of the correlation of the known causative gene with the overall LGMD2 gene set is highly significant relative to the mean number of genes (n = 72) in each 6 Mb interval (p-value = 1.24e-09). As an example, 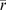-values surrounding the LGMD2J locus on 2q24 for which the known mutated gene is *TTN* are shown in Figure 3, which demonstrates the strongest correlation with *TTN* relative to all other probesets mapping to the 6 Mb interval. Since most of the genes involved in LGMD2 should be expected to play a role if expressed in muscle, the positive correlation is largely based on co-expression within muscle tissues and thus other muscle specific genes may also be highly correlated and within the interval. It is important to note that the success of UGET in identifying disease genes is inversely related to the size of the candidate interval, as larger intervals are more likely to contain multiple genes involved in the BP of interest which to not contribute to the disease of interest.

We applied this method to see if the tool was able to highlight genes involved in primary microcephaly, which is clinically diagnosed when an individual has a head circumference more than three standard deviations below the mean with no apparent biological or environmental cause. From a review of the Online Inheritance of Man [39], six loci have been mapped, and four genes identified: *ASPM, CENPJ, MCPH1, CDK5RAP2* thus far. The purpose of this test was to confirm that the methods used in our LGMD2 trial would effectively identify genes for a completely different and highly genetically heterogeneous phenotype, as well as to see if the method was robust enough to identify the known gene given a much smaller profile for comparison. In 75% (3/4) of cases the most correlated gene with the other known microcephaly genes was correctly identified from a 6 Mb linkage region surrounding that gene. Thus, for diseases that are genetically heterogeneous, once a small number of the identified genes is known, rank ordering other genes is a viable strategy.

**Figure 4:**
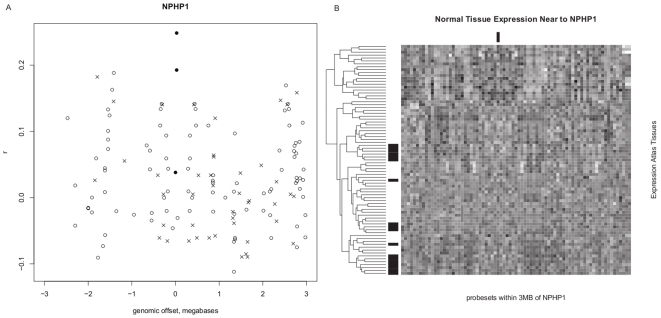
Gene correlations to a list of Joubert syndrome-associated genes within a 6 megabase region surrounding the location of an associated gene. **A**: The genomic position (x-axis) of probesets within a 6 megabase region centered at the location of a *NPHP1*, a gene known to be associated with Joubert syndrome, is plotted versus the Pearson correlation coefficient 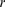 (y-axis) to a profile created from probesets of all other genes known to be associated with Joubert syndrome (excluding *NPHP1*) across 11,636 HG-U133_Plus_2 microarrays. Solid circles: probesets targeting *NPHP1*, 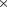: probesets not designed to target a known gene, open circles: other probesets across interval for known genes. **B**: Probeset positions in ascending genomic order (x-axis) versus tissue (y-axis) from the GNF Expression Atlas 2 are presented as a tissue-clustered, column-scaled heatmap. Black = low expression, white = high expression. Black bars in the margin indicate brain tissue rows, and the column representing Joubert syndrome-associated gene *NPHP1*.

We applied the scanning method to Joubert syndrome (JBTS), which is a genetically heterogeneous neurodevelopmental disorder characterized by the ‘molar tooth sign’ demonstrating hypoplasia of the cerebellar vermis and associated with developmental delay and various physical malformations. Eight linkage regions for Joubert syndrome have been identified (JBTS1-JBTS8). Five of these have had the associated gene in the region identified (JBST1 = *INPP5E*; JBTS3 = *AHI1*; JBTS4 = *NPHP1*; JBTS5 = *CEP290*; JBTS6 = *TMEM67*; JBTS7 = *RPGRIP1L*) [45], [46], [47], [48], [49], [50], [51], [52], [53], [54] while JBTS2 has so far only been linked to a 17 Mb centromeric region of chromosome 11 [50], [55], [56], [57], respectively. The purpose of this third test was to provide another instance of reproducibility of results and determine if we could make a prediction as to the identity of the genes in remaining linked regions JBTS1 and JBTS2 for which a gene has not yet been identified. We were able to correctly prioritize 67% (4/6) of the five genes known to be associated with JBTS as the first ranking candidate in a 6 Mb interval surrounding each. *AHI1* was ranked fourth out of 89 genes in its 6 Mb surrounding interval, and *INPP5E* was the 19^th^ of 98 genes. Both are positively correlated with the other known genes, but are not strongly highlighted indicating that the approach will not always be successful. An example of a successful identification is shown of the plot of 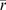-values surrounding *NPHP1* is given in Figure 4A. We also show data from the Gene Expression Atlas [17] for the same region in Figure 4B, demonstrating that *NPHP1* could not be identified merely by scanning this region for brain-specific or even brain-expressed genes. Thus, similar to the increased numbers of genes identified as cartilage-selective from the un-annotated arrays, there are subtle gene expression signals that are possible to identify simply from large scale data that preserve information about the similarity of gene expression in a variety of conditions (that are unknown to us) but remain informative for gene characterization. We examined JBTS2, which is a centromere-spanning 17 Mb region on chromosome 11 between markers D11S1915 and D11S4191. The best candidate based solely on the expression data for JBTS2 is *AGBL2*. However, this is a large region and there are other highly correlated genes on both sides of the centromere.

To assess the broader utility of UGET analysis, we examined ten additional genes identified as contributing to neuropsychiatric disorder within candidate genomic intervals. As autism is a widely studied common neuropsychiatric disorder with high heritability [58], [59], we postulate the expression patterns of genes likely contributing to autism [60] are broadly relevant neuropsychiatric disorders due to shared biological processes (central nervous system development and function). We thus used an autism-related expression module to rank genes within candidate regions using UGET (Table S1). In each case, the rank of genes within the candidate genomic interval was highly correlated with the gene known to contribute to the disorder, identifying the known gene as the first or second most highly correlated gene in five out of ten intervals (Table 2). Mean expression correlation scores for all ten genes studied were greater than one standard deviation above the mean for all genes, and greater than two standard deviations above the mean for eight out of the ten genes. While this analysis is not exhaustive, it strongly supports UGET as a powerful and broadly applicable candidate gene prioritization tool for complex disorders.

**Table 2:** Evaluation of expression-based candidate gene prioritization tools.

Disease Gene	Interval (Mb)	Disorder & Evidence	Total Genes	UGET		Endeavor		GeneDistiller	
				Rank	Genes	Rank	Genes	Rank	Genes
DOC2A	chr16:29.5–30.2	AutismRare Variant [62]	30	2	22	7	26	1	29
SEZ6L2	chr16:29.5–30.2	AutismAssociated Variant [62]	30	5	22	1	26	2	29
CACNA1G	chr17:20.1–46.1	AutismAssociation [63]	452	14	396	129	357	24	324
DOCK4	Chr7:106.2–118.6	AutismAssociation & CNV [64]	43	3	41	2	36	2	34
SYNJ1	chr21:30.5–46.9	Bipolar DisorderAssociation [65]	176	1	133	1	115	2	115
CACNA1A	chr19:4.9–18.2	FHMMonogenic Sub-type [66]	341	2	306	1	230	8	233
NTRK3	chr15:83–96.3	MDDAssociation [67]	63	1	58	15	45	4	61
ATP1A3	chr19:43.4–47.3	RDPMonogenic Sub-type [68]	112	2	103	15	79	2	90
PRKCA	chr17:54.9–68.4	SchizophreniaAssociation & Rare Variants [69]	89	4	82	15	74	4	81
NOS1AP	Chr1:156.2–161.8	SchizophreniaFunctional Common Variant [70]	110	5	88	3	78	2	66
TRPV4	chr12:107–119	ADBIBD Linkage & Functional Evidence [7]	149	1	72	27	85	5	103
ACAN	chr15:72–89.4	SEMDIBD Linkage & Genetic Evidence [8]	269	1	173	1	148	4	128

Genes within 11 mapped genetic intervals for 10 neuropsychiatric and two skeletal disorders were retrospectively ranked by UGET, Genedistiller, Endeavor by gene expression correlation to either known disease genes or genes which play important roles in an associated biological process. Grey boxes indicate that the disease gene was ranked in the top five candidates using the tool. Abbreviations: IBD = Identity-by-decent; CNV = Copy Number Variant; FHM = Familial hemiplegic migraine; MDD = Major Depressive Disorder; RDP = Rapid-onset dystonia Parkinsonism; ADB = Autosomal dominant brachyolmia; SEMD = Spondyloepimetaphyseal dysplasia; ATD = Asphyxiating thoracic dystrophy; SRP = Short rib polydactyly syndrome.

Using the 10 neuropsychiatric disorder intervals and the 3 skeletal dysplasia intervals mentioned above with the seed lists mentioned in Tables S2 and S1, respectively, we attempted to assess the relative efficacy of UGET compared other well used gene expression prioritization tools (GeneDistiller and Endeavour). The known disease gene was identified as a top 5 candidate gene in 11/13 disorders using UGET, 10/13 disorders using GeneDistiller, and 5/13 disorders using Endeavor. In the skeletal disorders, where the gene defect is in a cartilage-selective gene expressed in a rarely characterized tissue (i.e. cartilage growth plate), UGET outperformed these other tools.

### Human Disease Network

In order to determine the generality of the approach, we selected human disorders from a broad array of disease classes [61]. We selected 43 examples of disorders on the basis of the largest number of genes affiliated with each trait, while attempting to sample both Mendelian disorders and some more complex disorders such as hypertension. We then compared the Pearson correlation coefficients between all genes that are associated with a given disorder (intra-disorder correlation) to the Pearson correlation coefficients between these genes with all other genes in U133_2.0 arrays (extra-disorder correlation), and the results are shown in Table 3. In total, 36 of 43 (84%) had detectable enrichment in intra-disorder correlation with a p-value less than 0.05. Seven disorders had no such enrichment including Hirschsprung disease, Charcot Marie Tooth disease, Holoprosencephaly, Long QT syndrome, Spondyloepiphyseal dysplasia, and Nephronophthisis. This would indicate that the genes responsible for these syndromes are generally not co-expressed across diverse tissue types. Conversely, genes, mutations in which lead to cardiomyopathy, are very highly correlated with each other (p = 6.4×10^−146^) indicating that a restricted pattern of expression is common for this group of disease genes. The strongest mean intra-disorder correlation observed was for the genes causing Complement Component Deficiency, Ehlers-Danlos Syndrome and myopathy which all have mean intra-disorder correlation of over 0.26, which are highly significant and also are consistent with high expression in perhaps a restricted tissue or cell type. Included in this analysis were several common and clearly genetically complex disorders including obesity, hypertension, diabetes mellitus, schizophrenia, rheumatoid arthritis, and asthma. In all of these complex disorders genes that cause the disorder are substantially more likely to be correlated with the other known genes. This has the potential for providing important orthogonal associative information about individual genes in complex disorders in general, as genome wide association studies and rare variant searches are highly prone to false positive detections. Thus, there is strong evidence that genes that cause a given disorder are likely to be more similarly co-expressed across diverse gene expression experiments that UGET is detecting. To access this information more simply, a search tool which retrieves all of the genes linked to a given disorder has been created on the UGET website such that an investigator can select from the human disorder names and retrieve those genes mapping to that disorder for input into UGET.

**Table 3:** Disease genes within a classification are more strongly correlated with each other than with genes not linked to the disease.

Disorder Name	No. of Genes	No. of Probesets	Mean Intra-disorder Correlation	Mean Extra-disorder Correlation	Signif. of Enrichment
Epiphyseal_dysplasia	6	10	0.196	−0.005	1.9E-05
Osteoporosis	6	23	0.059	−0.007	1.3E-06
Amelogenesis_imperfecta	4	9	0.070	0.018	2.6E-02
Osteopetrosis	4	10	0.087	0.011	3.1E-03
Leukemia	37	162	0.024	0.016	5.0E-12
Colon_cancer	34	88	0.029	0.015	1.3E-08
Cardiomyopathy	25	68	0.155	0.009	6.4E-146
Hypertension	12	23	0.031	−0.001	5.2E-07
Long_QT_syndrome	7	13	0.019	0.012	5.6E-01
Ehlers-Danlos_syndrome	9	31	0.270	−0.018	7.5E-54
Rheumatoid_arthritis	8	25	0.075	0.021	2.4E-14
Epidermolysis_bullosa	11	25	0.236	0.002	6.5E-47
Ectodermal_dysplasia	8	18	0.096	0.016	6.9E-11
Holoprosencephaly	5	4	−0.008	0.015	3.9E-01
Deafness	41	96	0.016	0.008	8.8E-05
Diabetes_mellitus	27	54	0.025	0.014	3.4E-04
Hirschsprung_disease	7	14	0.010	0.002	2.5E-01
Hemolytic_anemia	10	21	0.119	0.000	2.6E-15
CCD	13	19	0.321	−0.010	3.4E-35
SCID	8	18	0.242	0.018	1.5E-30
Cent. Dis. Glycosylation	13	22	0.127	0.009	7.9E-17
Porphyria	6	26	0.073	0.008	3.5E-09
Fanconi_anemia	11	27	0.188	0.021	6.4E-37
Zellweger_syndrome	11	27	0.101	0.011	6.6E-27
Bardet-Biedl_syndrome	8	16	0.137	0.007	1.1E-08
Usher_syndrome	8	15	0.048	0.018	4.7E-03
Hermansky-Pudlak_synd	7	19	0.095	0.013	7.5E-11
Muscular_dystrophy	18	39	0.157	0.008	2.1E-66
Myopathy	10	15	0.264	0.005	2.8E-13
Mental_retardation	24	50	0.043	0.017	3.9E-10
Charcot-Marie-Tooth	18	36	0.018	0.008	9.0E-02
Spinocereballar_ataxia	13	27	0.061	0.022	3.8E-04
Leigh_syndrome	12	24	0.151	0.003	8.6E-18
Obesity	21	42	0.013	0.004	8.4E-04
Retinitis_pigmentosa	30	51	0.079	0.021	1.2E-26
Cataract	15	18	0.120	0.025	3.0E-09
Schizophrenia	9	20	0.082	0.012	1.2E-06
Renal_tubular_acidosis	5	11	0.034	0.002	4.3E-02
Nephronophthisis	4	12	0.006	0.010	8.1E-01
Asthma	13	24	0.039	0.004	4.0E-10
Cent_hypoventilation_syn	5	10	0.040	0.010	1.5E-02
Brachydactyly	5	10	0.011	−0.003	5.5E-02
Spondyloepiphyseal_dyspl.	5	9	0.003	0.015	5.8E-01

No. of genes: total number of genes in U133_2.0 arrays identified that are associated with a disorder name (44); No. of probesets: total number of probesets mapping to the genes; Mean Intra-disorder Correlation: mean Pearson correlation coefficient for all disease genes; Mean Extra-disorder Correlation: mean correlation between disease genes and all other genes in U133_2.0 arrays; Significance of Enrichment: t-test p-value comparing intra-disorder correlation with extra-disorder correlation.

## Discussion

We describe here the creation of a new web-accessible gene-gene correlation resource, and demonstrate the power and utility of a large collection of gene expression microarray data for functional gene discovery and for prioritizing genes for mutation analysis within linkage regions.

We first expanded a list of known cartilage-selective genes in mouse and humans. Within this process, 7 genes (out of 10) all with poor or little annotation were validated and demonstrate cartilage selective expression. Two novel genes tested, *SDK2* and *FLJ41170*, are very selectively expressed in fetal cartilage. The power of a large dataset is realized with this example as rare tissues (e.g.cartilage or fetal cartilage) and many novel genes (e.g. *FLJ41170*) are not present in Genesapiens. In addition, it is remarkable that despite the relatively small number of human fetal cartilage gene arrays in the public domain (<14 vs. 10,000 arrays), UGET is remarkably sensitive even when it comes to genes expressed in few tissues. Similarly, UGET can be used iteratively by scientists to identify genes with similar expression profiles for a variety of patterns in order to identify genes that may be involved in specific biological processes.

By simple use of UGET, the correct retrospective identification of the known causal genes within linkage regions for several unrelated disease phenotypes was demonstrated in the majority of cases. This simple application of the tool is in itself a highly powerful strategy because there are many more linkage regions reported than causal genes, yet there are many disease genes identified for a similar phenotype. Thus, we can effectively leverage current knowledge to prioritize genes within the many known linkage intervals. From our small number of examples, our evidence would indicate that, if there are at least a few known disease genes to use to create a profile of interest, the highest or second highest correlated gene will be the mutant gene at up to 80% of the loci. This approach is broadly applicable to more heterogeneous traits such as neuropsychiatric disorders, with 50% of previously identified genes within candidate intervals ranking either highest or second highest correlated gene using an autism-related expression module. A subset of genetically heterogeneous disorders however, has no strong correlation of expression between the multiple genes causing specific traits (for example, Hirschsprung's Disease). We note that the general process of gene prioritization applies to the entire genome and may point to strategies in the absence of linkage knowledge as well for rare Mendelian disorders. Likewise, the genetic causes of highly complex disorders such as autism or schizophrenia are certain to be numerous, but as true disease genes are identified in these disorders and others, gene-gene correlation analyses should be applied to prioritize additional genes in the absence of linkage or genome-wide association signals. We expect the tool to be generally useful in a wide variety of human disease areas and to expedite the gene discovery process. While there are other gene expression prioritization tools and other prioritization approaches (e.g. via interactome data and literature based) that are also successful, the data suggest that UGET is a robust tool especially when the genes and biological processes are better defined in rare or more complex datasets. In this regard, UGET takes advantage of the vast Celsius database which provides additional insight not possible using smaller, more discrete, but defined annotated datasets.

The tool is web accessible and easily searchable by scientists seeking to identify genome-wide gene-gene correlations. Data are returned as html lists for rapid perusal or as tab delimited text files available for download. The power and versatility of this resource initially surprised us and will be able to grow in power as microarray data accumulate. One of the powers of this approach is that the entire pipeline of methods used to assemble the correlation matrix is completely metadata independent – only the genomic alignment of probe sequences and the quantitative measurements made by the microarray were used. This results in a dataset that is very heterogeneous reflecting the diverse set of human experiments ongoing in the community. It is composed from microarray data generated from thousands of individual experiments by hundreds of individual scientists, with each experiment using different biological materials and different hybridization conditions and protocols. We conclude that the volume of data assembled here is sufficient and does not appear to have systematic biases based on site of origin of the data or differences in the method of data generation to mask true gene-gene correlations due to differences in microarray protocols.

A variety of analyses presented here have established that rank ordering of genes within a linkage interval using UGET is a successful approach in many cases. Generally, UGET is highly successful in ranking candidates when the disease gene has an expression pattern specific to the biological process being studied. Alternatively, genes involved in multiple biological processes (or genes not involved in the disease BP in normal individuals) may not rank as highly. As an example, *AHI1* was ranked fourth best candidate gene out of 89 genes when UGET was used to retrospectively identify known Joubert Syndrome (JBST) genes. *AHI1* is expressed in both central nervous system tissue and primitive hematopoietic cells, which diluted the strength of co-expression correlation to other JBST genes. This demonstrates key limitations in the application of the guilt by association approach to disease gene identification. False negative results will occur in such cases. This lessens the utility of UGET (and other methods such as Endeavour and SUSPECTS) to providing a specific type of biological insight. This limitation also leads to false positives arising if genes within a candidate interval are specifically expressed in the BP of interest but do not contribute to disease. Despite these limitations, in the majority of retrospective cases presented here (including *AHI1*), UGET analysis rank ordered candidate genes such that sequentially sequencing the most highly correlated genes would identify the known disease gene more far efficiently than sequencing all genes within the candidate interval. Thus, the limited scope of biological insight provided by UGET is nonetheless highly informative for human disease gene discovery.

While we demonstrate the utility of this tool particularly for human gene identification, the correlation matrices have been constructed for the entire Celsius dataset and are available for search within 14 different species across 41 different array designs. We note that the scale available through this resource is unprecedented and is the result of ignoring differences in annotation approaches by scientists. In general the genomics community has placed high value on the annotation information such that publication policies typically require metadata to be deposited concomitantly with assay measurements. However, without truly representing the vast diversity of experiments performed is daunting and not implemented yet. While annotation is useful and in some cases necessary for some supervised analysis, the practice of insisting on detailed metadata in the process of making raw microarray data available may actually limit the amount of raw data deposited. For instance, scientists have no strong motivation to provide annotation information on experiments that do not become part of the published experiment and thus these data will be excluded from repositories. In contrast to the annotation-centric efforts of microarray repositories, the Celsius database can import CEL data without annotation data and the work shown here demonstrates the enormous potential power of growing these data further. We recommend the deposition of all CEL files into public repositories or directly into Celsius to expand our ability to detect gene-gene correlations.

## Materials and Methods

### Data and Data Cleaning

We retrieved all RMA-processed gene expression data for the HG-U133_Plus_2 array design (n = 12,826 arrays) from the Celsius microarray data warehouse [17], [32] and denote the 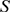 = 12826 (arrays) 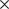 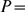
54675 (probesets) matrix as 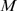. A cursory examination of 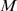 revealed that there were aberrant arrays present and that these arrays would have a negative impact on any downstream analyses and thus needed to be removed in a systematic manner. There appeared to be at least 3 groups of aberrant arrays:

1. arrays with extremely high gene expression values across many probesets.
2. arrays with extremely low gene expression values across many probesets.
3. arrays with dissimilar expression values for two probesets reputedly measuring the same gene.

We sought to systematically remove these arrays from the gene-gene correlation calculations. Group 1 and 2 arrays were easiest to identify for exclusion. We calculated the mean expression value of all probesets for each array, then calculated the mean and standard deviation of a 10% trimmed distribution of those means. The trimmed means themselves had a mean of 231.1 and a standard deviation of 21.0. There were 726 arrays with mean expression value more than 3 standard deviations away from the mean of trimmed means. These were primarily dim arrays (n = 711) but there were also excessively bright arrays (n = 15). These arrays were removed from further consideration, leaving matrix 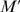 with 12,100 arrays and 54,675 probesets.

Group 3 arrays were slightly more difficult to find. To identify them, we exploited the fact that, via NetAffx [34], Affymetrix publishes a probeset 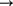 gene symbol mapping for their array designs. We assumed that pairs of probesets designed to target the same gene were more likely to be linearly related than randomly selected pairs because they were targeting the same gene, and that these relationships could be used as a starting point to identify inconsistent arrays.

There are 19,632 unique gene symbols from the NetAffx HG-U133_Plus_2 gene annotation. Of these, there is a subset 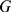 (n = 10,433) for which there were two or more probesets. We constructed 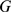 groups, each corresponding to a single gene symbol, i.e.: *g1 = p_g1_ 1,…,p_g1_n,…,g_G_ = p_gG_ 1,…, p_gG_n*. Then, for each 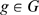, we performed a linear regression of *log_10_(signal)* for all possible probeset pairs *p_g_A,p_g_B* (n = 38,682). Examination of the probeset pairs with the largest value of 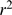 revealed that the majority were control probesets that targeted spike-in sequences that are added as part of the microarray hybridization for quality control. Thus, we concluded that using the built-in control probesets was a robust way to identify aberrant arrays. We performed 62 multiple regressions, allowing each control probeset to be the response variable once. In the context of a single regression if an array's residual was, relative to all other arrays' residuals, more than 3 standard deviations away from the line, we incremented a counter for that array. After performing all 62 regressions, all arrays that were observed more than 3 standard deviations more than 5% of the time (n = 464) were removed from further consideration, leaving a matrix 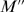 with 11,636 arrays and 54,675 probesets. Outlier frequencies per array are shown in Figure 1.

### Correlating Genes

Subsequent to filtering out aberrant arrays from out dataset, we used the 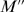 matrix to calculate 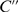, a 54675 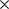 54675 matrix of Pearson correlation coefficients for every pair of probesets (Equation 1). 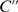 was used in all results presented in Section 4.
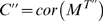
(1)


### Annotating Genes

For each probeset 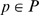 on the HG-U133_Plus_2 array design, we retrieved and sorted in descending order *r = C″_p_*. We took 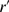, the derivative of 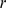, and used the R Bayesian Change Point *bcp* to identify 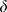, the index of the largest value of 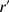 that preceded a mostly-linear portion of the curve. The subset of probesets where 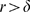 were defined as 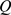, and used as input to the *hyperGTest* function of the *GOstats* package of Bioconductor [36] to test for enrichment of Gene Ontology (GO) Biological Process (BP) annotations in a gene set. *hyperGTest* produced a set of predicted gene annotations 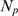 for each 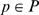 based on the annotation of neighbors 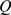. We applied Bonferroni correction to the p-values associated with each prediction by multiplying each p-value by the total number of predictions made for the corresponding probeset. We used these corrected p-values from predicted annotations 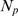 that were known to be non-computationally assigned from the *hgu133plus* package of Bioconductor [36] to establish a conservative cutoff, below which predicted annotations should all be high quality.

### Analyzing Linkage Regions

For a given phenotype, a group of known genes 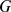 for which mutations have been described that lead to a specific phenotype were retrieved from previous publications and online databases. The list of genes was transformed to a list of probesets 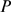 present on the HG-U133_Plus_2 array design using the gene symbol 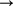 probeset mapping available from NetAffx [34]. All disease causing probesets 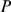 mapped to a unique location in the genome within a 6 Mb interval termed *A* around the known causative gene were identified by finding the center point of each probeset's alignment to UCSC March 2006 (hg18) version of the human genome [25]. Each region in 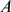 was then mapped to a list of all HG-U133_Plus_2 probesets 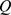 aligned to that region. Then, for each 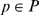, a QxG(GEg)lab was retrieved from 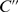 (Section 3.1.3), and row-summarized to produce a 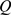-length vector 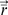 of mean correlation coefficients to 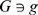.

### Analysis of Human Disease Genes

Genes for LGMD2, Joubert Syndrome, and Microcephaly were selected based on literature search/OMIM classifications as of September 2008. Disorder-gene mapping relied on affiliation [61]. All genes identified as causing 43 disorders, were first mapped to the Affymetrix U133_2.0 array to determine if a probeset existed for each gene. All probesets mapping to each gene were retrieved based on information at NetAffx. For each disorder, gene-gene correlation coefficients within a disorder causing gene list were calculated as described above except that for genes with multiple probesets, the correlation coefficients between the probesets of the same gene were excluded. We name them Intra-disorder correlation. As a general rule of thumb, probes with a mean Intra-disorder correlation ≥0.1 are considered nominally correlated. Gene-gene correlation coefficients between this gene list and all other genes in U133_2.0 arrays were also calculated. We name them Extra-disorder correlation. A two sample t-test was then performed comparing these two groups of correlation coefficients.

### qRT-PCR

One microgram of RNA from seven tissues (adipose, brain, kidney, ovary, heart, small intestine, and liver) in the FirstChoice® Human Total RNA survey panel (Ambion) was reverse transcribed using a high-capacity cDNA archive kit (ABI) and random primers. For cartilage, RNA from three independent cartilage samples was pooled and reverse transcribed. Amplification reactions were performed in duplicate using 50 ng of each cDNA. Thirty-five cycles of amplification were carried out in an ABI 7300 using the validated QuantiTect Gene Expression Assays and SYBR Green PCR kit (Qiagen). To assess specificity, amplification products were subjected to melting curve analysis and gel electrophoresis. The 2- [delta] [delta]Ct method was employed to calculate relative amplification. This was performed using an average of endogenous references (*18S*, *GAPDH*, and *HPRT1*) to improve normalization across the panel of tissues used. For genes where no amplification was detected in a tissue, a Ct value of 35 was assigned, reflecting the maximum number of cycles carried out.

## Supporting Information

Table S1 Representative genes are listed in rank order of mean similarity to the human cartilage-selective seeding profile identified in Figure 1 and represented in Column 1. Column 6 identifies genes, labeled with an X, which can result in skeletal abnormalities in humans [32]. Column 7 identifies associated mouse phenotypes summarized from Mouse Genome Database [33] (“N/A” indicates targeted model not available; “-” targeted mouse model has no skeletal phenotype yet). Column 8 indicates genes that were similarly identified in the mouse from Affy 430 2.0 whole genome array data as being in the top 100 cartilage selective genes. Column 8 indicates genes which are previously identified in Funari et al., 2007 et. al. as cartilage-selective. Column 9 indicates genes identified in Funari et al., 2007 as enriched in cartilage using data from the U133 2.0 platform and the first 200 ranked genes with a false discovery rate of zero using Significance of Microarray (SAM) analysis.
(0.04 MB XLS)

Table S2 To identify an autism-related expression model, the 26 genes identified as “probable” or “promising” in a recent review of autism genetics were used as both ‘training’ and ‘test’ sets in UGET. An expression module of 25 probes representing 13 genes (listed in this table) was identified as both highly inter-correlated and highly correlated to the total list of 26 “probable” or “promising” autism genes, and is thus deemed ‘autism-related.’ This module was used as a training set to assess the mean co-expression correlation between the module and all 54,613 probes on the Affymetrix U133A_2.0 Human Gene Expression Microarray. The most highly correlated probe mapping to a given gene was selected as representative for that gene.
(0.04 MB DOC)
