# Supplementary material for: Disease Gene Characterization through Large-Scale Co-Expression Analysis
Source: PLoS One. 2009 Dec 31;4(12):e8491. doi: 10.1371/journal.pone.0008491 (PMC2797297; doi:10.1371/journal.pone.0008491)
Supplement: Table S2 — To identify an autism-related expression model, the 26 genes identified as “probable” or “promising” in a recent review of autism genetics were used as both ‘training’ and ‘test’ sets in UGET. An expression module of 25 probes representing 13 genes (listed in this table) was identified as both highly inter-correlated and highly correlated to the total list of 26 “probable” or “promising” autism genes, and is thus deemed ‘autism-related.’ This module was used as a training set to assess the mean co-expression correlation between the module and all 54,613 probes on the Affymetrix U133A_2.0 Human Gene Expression Microarray. The most highly correlated probe mapping to a given gene was selected as representative for that gene. (0.04 MB DOC) [file pone.0008491.s002.doc]

| Gene | Probe ID |
| --- | --- |
| AHI1 | 221569_at |
| CACNA1C | 242973_at |
| CACNA1C | 238636_at |
| CADPS2 | 219572_at |
| CNTNAP2 | 219300_s_at |
| CNTNAP2 | 215145_s_at |
| GABRB3 | 229724_at |
| GABRB3 | 205850_s_at |
| GABRB3 | 227690_at |
| GABRB3 | 227830_at |
| GRIK2 | 213845_at |
| GRIK2 | 1560265_at |
| MECP2 | 202616_s_at |
| MECP2 | 202617_s_at |
| NRXN1 | 228547_at |
| NRXN1 | 209914_s_at |
| NRXN1 | 209915_s_at |
| NRXN1 | 216096_s_at |
| RELN | 205923_at |
| SHANK3 | 227923_at |
| SLC25A12 | 203340_s_at |
| SLC25A12 | 203339_at |
| TSC1 | 209390_at |
| UBE3A | 234163_at |

To identify an autism-related expression model, the 26 genes identified as "probable" or "promising" in a recent review of autism genetics were used as both 'training' and 'test' sets in UGET. An expression module of 25 probes representing 13 genes (listed in this table) was identified as both highly inter-correlated and highly correlated to the total list of 26 "probable" or "promising" autism genes, and is thus deemed 'autism-related.' This module was used as a training set to assess the mean co-expression correlation between the module and all 54,613 probes on the Affymetrix U133A_2.0 Human Gene Expression Microarray. The most highly correlated probe mapping to a given gene was selected as representative for that gene.
